# Machine Learning in the Development of Adsorbents for Clean Energy Application and Greenhouse Gas Capture

**DOI:** 10.1002/advs.202203899

**Published:** 2022-10-26

**Authors:** Haoxin Mai, Tu C. Le, Dehong Chen, David A. Winkler, Rachel A. Caruso

**Affiliations:** ^1^ Applied Chemistry and Environmental Science School of Science STEM College RMIT University Melbourne Victoria 3001 Australia; ^2^ School of Engineering STEM College RMIT University GPO Box 2476 Melbourne Victoria 3001 Australia; ^3^ Monash Institute of Pharmaceutical Sciences Monash University Parkville VIC 3052 Australia; ^4^ School of Biochemistry and Chemistry La Trobe University Kingsbury Drive Bundoora 3042 Australia; ^5^ School of Pharmacy University of Nottingham Nottingham NG7 2RD UK

**Keywords:** covalent–organic frameworks, hydrogen, intermetallics, metal–organic frameworks, porous carbons, porous polymers networks, zeolites

## Abstract

Addressing climate change challenges by reducing greenhouse gas levels requires innovative adsorbent materials for clean energy applications. Recent progress in machine learning has stimulated technological breakthroughs in the discovery, design, and deployment of materials with potential for high‐performance and low‐cost clean energy applications. This review summarizes basic machine learning methods—data collection, featurization, model generation, and model evaluation—and reviews their use in the development of robust adsorbent materials. Key case studies are provided where these methods are used to accelerate adsorbent materials design and discovery, optimize synthesis conditions, and understand complex feature–property relationships. The review provides a concise resource for researchers wishing to use machine learning methods to rapidly develop effective adsorbent materials with a positive impact on the environment.

## Graphical abstract

This review outlines basic machine learning methods, overviews their use in developing robust adsorbent materials, and provides a perspective on future advances in this research area. The application of machine learning to materials science will continue to bring to light innovative adsorbent materials with potential in future energy storage and conversion applications, gas separation, and greenhouse gas adsorption.

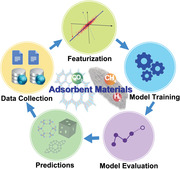

## 1 Introduction

The 2016 Paris agreement aimed to limit global warming to less than 2 °C by 2100, an essential target for addressing climate change.^[^ ^1^, ^2^ ^]^ To achieve this, the research community must develop innovative processes for generating clean energy and reducing greenhouse gas emissions.^[^ ^3^, ^4^, ^5^ ^]^ More environmentally friendly gaseous fuels with high energy generation efficiency, such as H_2_, are attractive alternatives to nonrenewable fossil fuels.^[^ ^6^, ^7^ ^]^ Although their gravimetric energy density is excellent, their volumetric energy density under normal temperatures and pressures is low, leading to storage and transport challenges.^[^ ^8^, ^9^ ^]^ Conventional technologies for storage and transport, like compression and liquefaction, require high pressures or low temperatures.^[^ ^10^, ^11^ ^]^ The environmental impact of greenhouse gases can be ameliorated by their capture and storage, or conversion to useful chemicals.^[^ ^12^, ^13^ ^]^ Currently, chemical absorption using amine aqueous solutions is the most common method of absorbing CO_2_. However, safety and cost issues related to corrosion, energy consumption, and amine loss remain.^[^ ^14^, ^15^ ^]^ Therefore, cheaper and safer technologies for storage and transport of gaseous fuels and the large‐scale separation and adsorption of CO_2_ are a high priority.

Among the solid adsorbents, porous materials such as metal–organic frameworks (MOFs),^[^ ^9^, ^16^, ^17^ ^]^ covalent–organic frameworks (COFs),^[^ ^18^, ^19^ ^]^ porous carbons,^[^ ^20^ ^]^ and zeolites^[^ ^21^ ^]^ have excellent H_2_, CH_4_, and CO_2_ adsorption abilities, low cost, scalable production, and tunable structural features. However, rapid development of these materials is hampered by two obstacles.^[^ ^16^, ^22^ ^]^ First, the structural and compositional space of these materials is vast, making full experimental exploration impossible. Second, the relationships between materials features and their desired properties (e.g., uptake capacity and selectivity) are complex and often nonlinear. This can make physics‐based computational modeling and experimental characterization to identify the most relevant features complicated, time‐consuming and expensive, hindering material innovation. More effective strategies must be developed to shorten discovery timelines for efficient adsorbents.

Data acquired from experiments and high‐throughput computation can be used to train machine learning (ML) models of the properties of porous materials that can be used to expedite the design, discovery, and optimization of adsorbents.^[^ ^23^, ^24^, ^25^ ^]^ Data‐driven ML methods such as neural networks (NN), support vector machines (SVM), and Bayesian methods can uncover complex relationships between porous materials structural, physicochemical, and process properties and useful properties such as uptake and selectivity.^[^ ^26^ ^]^ These models can make quantitative predictions of these properties for new porous materials yet to be synthesized.^[^ ^27^ ^]^ Given the exponential growth of research data, ML approaches are driving new materials discovery and elucidating complex feature–property relationships in large porous materials chemistry spaces.^[^ ^28^, ^29^, ^30^, ^31^ ^]^

This review provides a broad introduction to ML methods used for the design (using chemical intuition/skill or computational guidance to generate similar structures), discovery (screening unknown material spaces by high‐throughput experiments or computations), and optimization (modifying the structures of lead materials or the synthesis conditions to improve their properties) of functional materials for clean energy applications, such as adsorption and separation of H_2_ and CH_4_, and reduction of greenhouse gases. Recent reviews that combine data‐science and porous materials are available, some covering specific areas (e.g., high‐throughput methods, nanotechnology, and materials evolution), in which ML is a part of the strategy.^[^ ^32^, ^33^ ^]^ Other reviews are substantially ML focused, and potentially less relevant to materials scientists lacking strong data science backgrounds.^[^ ^24^ ^]^ As many readers may not be familiar with ML techniques, our review summarizes the application of ML to different types of adsorbent materials, introduces the most recent advances in adsorbent materials developed by ML‐assisted strategies, and provides readers with a practical guide to selecting ML methods for research on a wide range of adsorbent materials. More extensive, recent reviews on the applications of ML to materials design in general are available for interested readers.^[^ ^24^, ^34^, ^35^ ^]^ The focus here is on using ML methods to develop high performing, diverse adsorbent materials. Section 2 introduces key steps in applying ML methods to adsorbent materials. Recent examples of adsorbent design and optimization using ML are summarized in Section 3. The final section presents conclusions and provides a perspective on likely progress in this field in the short to medium term. We hope this review will stimulate and encourage the use of ML techniques to accelerate development of effective adsorbents, leading to improved renewable energy technologies.

## 2 Developing Machine Learning Models

**Figure 1:**
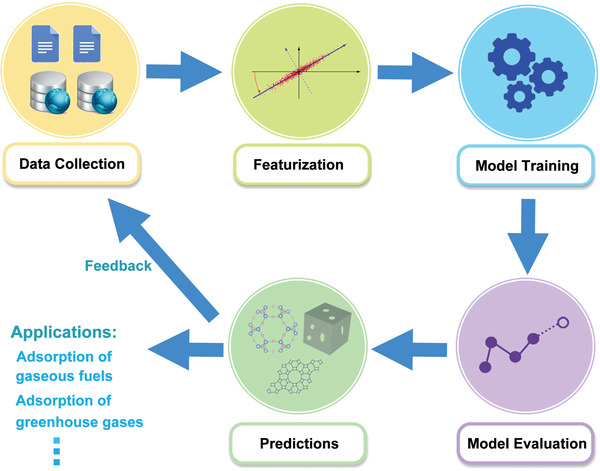
Workflow for applying ML techniques in the development of porous materials for gas adsorption. ML model construction includes data collection, featurization, model training, and model evaluation. The ML models are then used to make predictions. Predicted materials that are subsequently synthesized and assessed will be added to the original dataset for model improvement, and the materials with better properties will be progressed to practical applications.

ML uses training data and an appropriate algorithm to model diverse relationships in physical or biological systems.^[^ ^28^, ^36^ ^]^ It is an empirical alternative to complicated static and dynamic first‐principles electronic structure and molecular dynamics calculations and provides insight into nonlinear, multidimensional, feature–property relations. The process of constructing a ML model consists of four steps as follows (**Figure** **1**): 1) data collection, 2) feature generation and selection, 3) algorithm selection and mapping, and 4) model validation and prediction.

Data collection is extremely important for material informatics. Data must be reliable, of sufficient volume, low in noise and bias, and the modeled property should have a reasonable dynamic range of values (i.e., a model cannot be generated if all data points have similar properties).^[^ ^37^ ^]^

Data for ML model training include features (from molecular structures, physicochemical properties, experimental conditions of synthesis, etc.) and the target properties. Target properties for materials in the dataset are usually derived from laboratory and computational experiments.^[^ ^38^ ^]^ Models are most successful when they are trained on data with a wide range of properties—materials with poor properties as well as the successful materials.^[^ ^39^, ^40^ ^]^ A paucity of large, high‐quality datasets has necessitated the use of data collected from the literature, ideally compiled into data repositories.^[^ ^41^, ^42^, ^43^, ^44^ ^]^ These materials databases are important sources of curated data. Examples of databases for adsorbent materials include molecule databases (ChEMBL,^[^ ^45^ ^]^ GDB‐13,^[^ ^46^ ^]^ GDB‐17,^[^ ^47^ ^]^ ZINC,^[^ ^48^ ^]^ etc.), inorganic compound databases (Atomic‐FLOWLIB,^[^ ^49^ ^]^ Inorganic Crystal Structure Database,^[^ ^50^ ^]^ Materials Project,^[^ ^51^ ^]^ Open Quantum Materials Database, NOMAD,^[^ ^52^ ^]^ etc.), and databases for specific materials, such as the Cambridge Structural Database MOF subset,^[^ ^53^ ^]^ the Computation‐Ready, Experimental MOF Database,^[^ ^54^ ^]^ the International Zeolite Association (IZA) database, the Predicted Crystallography Open Database (mainly for silicates, phosphates, sulfates, zeolites, and fluorides),^[^ ^55^ ^]^ the NIST isotherm database (ISODB),^[^ ^56^ ^]^ and the Metal Organic Framework Database (MOFDB).^[^ ^57^ ^]^ Many of these databases contain materials structures, and some record important properties of materials. For example, the Materials Project has DFT‐calculated electronic properties available for a large number of materials. NIST ISODB and MOFDB are databases of adsorbents and isotherms.

Once sufficient data are collected, information about the materials must be converted into mathematical representations (descriptors, or features) suitable for training ML models. The quality and relevance of these representations play a major role in the quality of the subsequent model, accuracy of predictions of properties for new materials, and the ability to interpret the models in terms of chemistry (useful for deciding which material to synthesize next). The conversion of relevant attributes of materials into features is called featurization. The performance of ML models is optimum when the features used in training are most relevant to the property being modeled. Featurization is of critical importance to the quality and interpretability of the models generated. Ideally, materials and data scientists should work together to identify the features with the most promise.

A large number of features can be calculated or measured for adsorbent materials, and the importance of these features for a material is strongly context dependent.^[^ ^28^, ^31^, ^36^ ^]^ For example, experimental features (temperature, pH value, pressure, reaction time, and the amount of the reactants) would be used to construct models for synthesis optimization of adsorbent materials.^[^ ^58^ ^]^ Topographical features (pore size, volume, surface area, topological shape) and compositional features are frequently used when searching for adsorbent materials with the best texture and composition for gas uptake.^[^ ^59^ ^]^ Atomic features (e.g., atomic radii, mass, number of valence electrons) and electronic features (e.g., electronegativity, ionization energy, polarizability) are frequently used for selection of the coordinating metals of MOFs.^[^ ^35^, ^60^ ^]^ For MOF linkers, electronic features, such as bandgap, dipole moment, highest occupied molecular orbital (HOMO), lowest unoccupied molecular orbital (LUMO), and other structural features such as the Coulomb matrix,^[^ ^61^ ^]^ atom‐centered symmetry functions (ACSF),^[^ ^62^ ^]^ simplified molecular‐input line‐entry system (SMILES),^[^ ^63^ ^]^ Voronoi tessellations,^[^ ^64^ ^]^ and Smooth Overlap of Atomic Positions (SOAP),^[^ ^65^ ^]^ are used to describe their molecular structures.^[^ ^35^ ^]^ Distances between neighboring atoms are often used to describe or encode the local structure of adsorbent materials.^[^ ^66^ ^]^ Some physical coefficients, such as Henry's law constant, which is closely related to the adsorption isotherm, can be used as a feature or target property.^[^ ^67^, ^68^, ^69^ ^]^ In addition, energy‐based features, including Voronoi energy^[^ ^70^ ^]^ and energy histogram,^[^ ^71^ ^]^ are used to improve the model performance in the cases when the training set is small and the data diversity is high. Fanourgakis et al. developed a set of new features related to the MOF energy surfaces by inserting probe atoms of different sizes into the MOF, and found that model predictions were improved by using these features.^[^ ^72^ ^]^ We suggest that despite computational complexity, structural features and energy‐based features play more important roles in gas uptake prediction than electronic and atomic features,^[^ ^73^ ^]^ while topographical features and composition‐based features are frequently used due to their ease of calculation.^[^ ^74^, ^75^ ^]^ Therefore, there is a trade‐off between the computational complexity and model accuracy and interpretability. Combining multiple features provides a more accurate description of materials.

Although many features may correlate with target properties, the number of features must be limited to avoid overfitting and degradation of model predictivity by the presence of features of low relevance (noise).^[^ ^31^ ^]^ Large numbers of features also increase the complexity of the model, increasing the computation expense, compromising the prediction ability (optimally sparse models have the best predictive abilities), and making model interpretation more difficult.^[^ ^36^ ^]^ As a rule of thumb, the number of fitted variables in a model should be less than half of the number of the data points, preferably much less.^[^ ^36^ ^]^ Down‐selection and dimensionality reduction are two strategies often used to reduce the number of features. In down‐selection, statistical methods, such as the least absolute shrinkage and selection operator (LASSO) or random forest (RF), are used to assess the importance of the features, and the least important features can be discarded from the feature set.^[^ ^24^ ^]^ However, the performance of the down‐selection algorithms depends on the choice of hyperparameters. Dimensionality reduction is the alternative method to shrink the feature set. The principle of this strategy is to project the data points from a high dimensional feature space to a low dimensional feature space. As it is well known that sparser models have better generalization ability and interpretability,^[^ ^36^ ^]^ some features in these models may be highly correlated or do not strongly relate to the target property. During dimensionality reduction, these features are combined, and new features are generated. Therefore, relevant feature information is retained and only the redundant information is lost when an appropriate dimensionality reduction algorithm is used. The ability of models to predict the properties of new, unseen materials will be enhanced after such dimensionality reduction, thus the performance of the dimensionality reduction algorithm can be estimated by model evaluation. The most popular linear dimensionality reduction algorithms are principal component analysis (PCA) and linear discriminant analysis (LDA).^[^ ^76^, ^77^, ^78^ ^]^ The PCA method generates orthogonal features that decrease or remove correlations.^[^ ^79^ ^]^ This is an unsupervised projection algorithm, computing a group of orthogonal vectors (principal component) as new features, where the data point variances are maximum. In contrast to PCA, LDA looks for the orthogonal vectors on which the variances of the data points among different classes are maximum. Both PCA and LDA can simplify ML models and remove the issue of dimensionality, but they suffer from the assumption that the relationship between features and modeled property is linear, which is often not the case. Nonlinear projection algorithms, such as Isomap, Local Linear Embedding, Laplacian Eigenmaps, t‐distributed stochastic neighbor embedding (t‐SNE), and uniform manifold approximation and projection (UMAP) can perform nonlinear dimensionality reduction.^[^ ^80^, ^81^ ^]^ Among these algorithms, t‐SNE and UMAP have been extensively applied to data visualization and evaluating the domain of applicability of ML models on different datasets. Note that less relevant information is retained after some dimensional reduction methods, sometimes resulting in reduced model performance.

When the materials in a dataset are described by a relevant subset of features, an ML algorithm is chosen to train models using these data. The choice of algorithm depends on the nature of the dataset and problem to be solved. For example, supervised learning is used for gas uptake capacity prediction and adsorbent screening. In supervised learning, all the training data must be labelled by the property of interest.^[^ ^36^ ^]^ Unsupervised learning is preferred when the aim is to identify the patterns and trends in unlabelled data.^[^ ^93^ ^]^ The largest difference in the performance of ML algorithms is between linear and nonlinear methods. **Table** **1** summarizes algorithms commonly used in materials science.

**Table 1:** The characteristics and disadvantages of different ML algorithms and their applications to adsorbent materials

Algorithm	Purposes	Characteristics	Disadvantages	Examples
Linear regression	Regression	Simple and fast Good performance on small datasets Good interpretability	Poor performance when feature–property relations are nonlinear	[71, 75, 78, 82]
Logistic regression	Classification	Simple and fast Good performance on small datasets Easy to be updated with new data	Poor performance when feature–property relations are nonlinear Poor performance on high dimensional feature spaces	[83]
Kernel ridge regression (KRR)	Regression	Provide nonlinear solution	Slow on large datasets	[43, 84, 85]
k‐nearest neighbors (kNN)	Classification, regression	Simple principles Insensitive to outliers Nonlinear analysis Easy to be updated with new data	Numbers of neighbors (k) are defined by user Slow on large datasets Poor performance on biased samples	[75, 78, 86]
Naive Bayes	Classification	Fast Insensitive to missing data and irrelevant features Multi‐class predictions Easy to be updated with new data Good interpretability	Each feature should have independent and equal contribution to the outcome	[86]
Support vector machine (SVM)	Classification, regression	Available on high dimensional feature spaces Provide nonlinear solution Provide small‐sample solution Insensitive to outliers Global solution (no local minima issue) Good generalization ability	Slow on large datasets No general rule for choosing kernel function Only available for binary classification Sensitive to missing data Poor interpretability Tends to overfit models	[75, 78, 83, 86, 87]
Random forest (RF)	Classification, regression	Ensemble of decision trees Available on large datasets with high feature space dimensionality Can handle missing data Insensitive to outliers Good generalization ability Can evaluate feature importance (thus can be used in feature selection) Resistance to overfitting	High complexity Slow (when the number of decision trees is large) Each decision tree should be independent Biased for small datasets	[70, 75, 78, 83, 86, 88]
Extremely randomized trees (EXT)	Classification, regression	Similar to RF except using the whole original sample instead of bootstrap Choose cut points randomly instead of optimum split Less variance than RF Better generalization than RF	Generates more decision trees in the model than RF Larger bias than RF	[75, 88]
Gradient boosting trees (GBT)	Classification, regression	Ensemble of decision trees Can handle missing data Can evaluate feature importance (thus can be used in feature selection)	Sensitive to outliers Tend to overfit when the number of decision trees is large Slow for large datasets	[43, 75, 88]
XGBoost	Classification, regression	More accurate than GBT The in‐built regularization can prevent overfitting Can handle missing data	Slow for large datasets High space complexity	[88]
Neural network (NN)	Classification, regression	High accuracy Intensive learning ability Can store information in the network Robust to missing data and noise	No objective method for choosing architecture unless Bayesian regularized Poor interpretability Local minima issues	[43, 78, 83, 89]
Deep neural networks	Classification, regression	High accuracy Intensive learning ability Can store information in the network Robust to missing data and noise Can generate useful latent descriptors from simple molecular representations	Slow Many weights requiring large training datasets Poor interpretability High complexity Requires effective regularization to avoid overfitting	[90, 91, 92]
k‐means	Clustering	Simple and fast Unsupervised	Numbers of clusters (k) are defined by user Poor performance when the shapes of clusters are irregular Sensitive to noise	[78]
DBSCAN	Clustering	Numbers of clusters are obtained by the algorithm Can handle clusters with irregular shapes Can identify clusters and noise Unsupervised	Slow, especially on large datasets Poor performance when the clusters have very different densities	[23]

**Figure 2:**
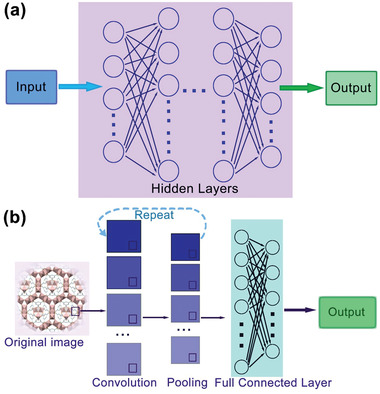
Representations of a) deep neural networks (DNN) and b) convolutional neural networks (CNN). The DNN comprises one input layer, one output layer, and a few hidden layers, each of the hidden layer contains multiple neurons, where the inputs were passed forward and errors backward to adjust the weights and bias of each node. The CNN is a class of DNN in which the hidden layers consist of several convolutional and pooling layers. The full connected layer is a traditional multilayer NN used to predict the value or the class of the images.

Among the nonlinear algorithms, NNs have been successfully applied to materials science, particularly for the development of adsorbent materials.^[^ ^24^, ^94^, ^95^, ^96^, ^97^, ^98^, ^99^, ^100^, ^101^, ^102^ ^]^ NNs are composed of an input layer, an output layer and interconnected hidden layers.^[^ ^103^, ^104^ ^]^ In each hidden layer, there are a series of units (neurons) containing nonlinear transfer functions that pass inputs forward and errors backward to allow the weights and biases of each unit to be adjusted. The number of hidden layer neurons depends on the nonlinearity of the problem to be solved. Simple neural networks usually contain a single hidden layer with relatively few neurons. A deep neural network (DNN) contains multiple hidden layers, each layer containing many neurons (**Figure** **2**a). DNNs have numerous applications in adsorbent materials science, including design of adsorbent materials with high gas adsorption rate and illustration of the underlying structure–adsorption relationships.^[^ ^90^, ^91^, ^105^, ^106^ ^]^ A subset of DNNs, convolutional neural networks (CNNs), are very useful for image recognition and analysis.^[^ ^104^ ^]^ Unlike other DNNs, the hidden layers of CNN consist of a number of convolutional and pooling layers (Figure 2b). The convolutional layer maps the input tensor to a feature map using multiple kernel filters then transmits the output to the pooling layer that performs downsampling and further convolution. The final feature map with more abstracted features is transmitted to the fully connected layer for regression or classification. For adsorbent materials investigations CNNs have been trained to extract the chemical and physical characteristics from their topology images or diverse spectra.^[^ ^107^, ^108^, ^109^, ^110^, ^111^ ^]^

Finally, the accuracy of the model predictions must be evaluated. For regression models, the error metrics are the coefficient of determination (*r* ^2^), root mean square error (RMSE), mean absolute error (MAE), and mean absolute percentage error (MAPE). The RMSE and MAE are preferred over *r* ^2^ values as they are not dependent on the number of data points and number of parameters in the model.^[^ ^112^, ^113^ ^]^ MAE values are less biased by one or two large outliers in the predictions than RMSE values. Likewise, MAPE is independent of scale and easy to interpret, but it will become infinite when there are actual values close to zero. For the classification models, accuracy, F1 score, geometric mean of recall and precision (G‐mean), and the area under the receiver operating characteristic curve (AUC) are the metrics widely used for scoring model performance. F1 score, G‐mean, and AUC are suitable for unbalanced classification models where one class is more highly represented than the other. By using these metrics, we can evaluate how accurately the models can predict the properties of the training data used to generate the model, and the test data that is not used in training. A good regression model should have the *r* ^2^ close to 1, while RMSE and MAE should be close to 0 on both training set and test set. For a good classification, the accuracy, F1 score, G‐mean and AUC should all be close to 1 on both training set and test set. A model is underfitting when the performance on the training set is poor, while overfitting is identified when good performance is obtained on the training set but poor performance on the test set. Both underfitting and overfitting can be avoided by increasing the size of the dataset, using fewer or greater relevance features, and modeling using linear and nonlinear ML algorithms.

Model interpretation is of critical importance in material research. Linear regression and decision tree‐based models are intrinsically interpretable and provide global interpretations. In some cases, however, interpretation can fail to capture true feature–property relationships. For example, a linear model cannot explain nonlinear relationships no matter how much regularization is carried out. To address these issues and allow interpretation of nonlinear models, new methods have been developed. A commonly used method is permutation feature importance, which estimates the importance of a feature by calculating the increases of the model error after permutating this feature.^[^ ^114^ ^]^ This method is fast, easy to understand, and gives global interpretation of features that span a wide range for nonlinear relationships, but it may be bias when there are correlated features, it cannot illustrate the effects of features on predicted values, and the results may lack reproducibility as it adds randomness in the calculation. Shapley additive explanation (SHAP) is another popular method in ML studies.^[^ ^115^ ^]^ SHAP can measure the contribution of each feature to the prediction of an individual sample. It attempts to generate global interpretations, usually spanning a range of values and signs due to the fact that feature importance is a local property for nonlinear models.^[^ ^116^, ^117^, ^118^ ^]^ SHAP can also give unreliable results when features are correlated, and thus the results should be scrutinized by domain specialists. For NN models, salience methods (e.g., class activation maps),^[^ ^119^ ^]^ attention masks,^[^ ^120^ ^]^ and partial derivatives (sensitivity analysis)^[^ ^121^ ^]^ are used to interpret these “black box” models. Interested readers can find more details about model interpretations in the recent review by Oviedo et al.^[^ ^122^ ^]^

Good practice in ML research requires good quality data.^[^ ^26^, ^36^ ^]^ All data used, especially that obtained from different sources, should be reproducible and comparable. To avoid overfitting, featurization must be applied to optimize the number of features.^[^ ^24^, ^26^, ^31^, ^36^ ^]^ Some features, such as structural and energy features, are more important for predicting gas adsorption for adsorbent materials than others, but the complexity of their measurement and calculation should also be considered. The research objective and nature of the structure–property relationship determine the selection of the ML model. Clearly, models with good predictivity and interpretability are preferred, while training time, size of training set, and domains of applicability (the range of properties in which the model makes reliable predictions) should also be considered.^[^ ^24^, ^36^, ^123^ ^]^ Using informative features improves model predictivity and interpretability. Selecting such features and model interpretation requires collaboration between materials scientists and data scientists. Ideally, ML models should be consistent with the known physicochemical theories or provide new insight for materials science.

## 3 Examples of Machine Learning Approaches for Adsorbent Materials

ML models can be used in materials science in three main ways: discovery, design, and optimization. In the development of adsorbent materials, high‐throughput first principles calculations can be useful to simulate some properties but are limited by the time and cost of the calculations. The gas adsorption capacity of an adsorbent material, one of the most important metrics for these materials in commercial use, is influenced by local properties such as structure, topology, and sorption sites for gas molecules; properties difficult to simulate by physics‐based methods on a large scale. However, ML is not only effective in predicting local properties but also in properties that are influenced by intrinsic and external factors (e.g., gas adsorption and selectivity).^[^ ^24^, ^28^, ^31^, ^124^ ^]^ ML models can be trained on the data generated by high‐throughput calculations to rapidly predict the target properties of related materials.^[^ ^30^, ^125^, ^126^ ^]^ New materials are best discovered using ML models with broad domains of applicability to screen large databases of real or hypothetical materials to identify candidates with potentially useful properties.^[^ ^37^, ^127^ ^]^ The domain of a model is the range of feature space and property space represented by the training data. The further away from the domain that predictions are made, the less accurate they will be. Moreover, target properties of materials can be improved by modifying the structure, composition, or other features that are suggested to have critical effects by ML models.^[^ ^39^, ^128^, ^129^ ^]^ In this section, ML applications to adsorbent materials are discussed, including metal–organic frameworks, porous carbons, zeolites, covalent–organic frameworks, porous polymers networks, and intermetallics.

### 3.1 Metal–Organic Frameworks

MOFs are a class of hybrid inorganic‐organic nanoporous materials formed by the self‐assembly of metal clusters and polydentate organic linkers as structural units that create open crystalline frameworks.^[^ ^17^ ^]^ Since their discovery, MOFs have garnered significant interest for a wide range of applications such as gas separation and storage, catalysis, sensing, nonlinear optics and as light absorbers due to their highly tunable nature.^[^ ^130^, ^131^, ^132^, ^133^, ^134^ ^]^ In principle, databases of millions of new MOF structures can be readily generated by systematic functionalization of the well‐known organic linkers, from which new MOFs with excellent target properties may be identified.^[^ ^135^, ^136^, ^137^ ^]^ However, finding an optimal MOF structure for a given application is challenging. For example, for Zn_2_(1,4‐benzenedicarboxylate)_2_(pyrazine) (ZBP), when the four symmetric substitution points in this compound are functionalized by a small library of 35 functional groups, there are a total of 35^4^ possible combinations of new MOF structures.^[^ ^138^ ^]^ It is impractical to locate the optimal MOF structures in such large databases using experiments or physics‐based computational methods. Therefore, ML methods can alleviate the computational burden by preselecting candidates with predicted high performance.^[^ ^40^, ^139^, ^140^, ^141^, ^142^, ^143^, ^144^ ^]^

The porous structure, large surface area, and tunability of MOFs provide exceptional performance in gas separation and storage, especially storage of hydrogen, carbon dioxide, and methane.^[^ ^133^, ^137^, ^145^ ^]^ Many new MOF structures with high gas uptake values and good selectivity have been discovered with the assistance of ML techniques. Fernandez et al. described a robust SVM classifier that rapidly identified promising MOFs for CO_2_ capture.^[^ ^146^ ^]^ A database of 324 500 hypothetical MOF structures was generated by combining 66 structural building units and 19 functional groups, of which 10% were randomly selected to form a training set. Atomic property‐weighted radial distribution function (AP‐RDF) descriptors were used to represent the atomic properties and electronic structural information of the MOF structures. A grand canonical Monte Carlo (GCMC) simulation was carried out to label the data points with the CO_2_ uptake values at 0.15 and 1 bar CO_2_ at 298 K.^[^ ^147^ ^]^ When screening a material space of 292 050 MOFs, the 0.15 bar classifier successfully identified 945 of the 1000 MOFs with the highest CO_2_ adsorption capacity in the dataset. As the properties of only 10% of the MOFs in the database needed to be calculated, and a high accuracy prediction was achieved, the ML approach proved useful for accelerated screening of large search spaces. Burner et al. developed a NN model to predict the CO_2_ uptake capacity and CO_2_/N_2_ selectivity of MOFs under low pressure.^[^ ^148^ ^]^ The model was trained on a dataset of 340 000 MOFs with over 1000 topologies. They found that the model had the best pe rformance when six geometric descriptors together with AP‐RDF and chemical motifs were used as features. The model identified 994 MOFs with the highest CO_2_ adsorption capacity from a test set of ≈70 000 MOFs.

**Figure 3:**
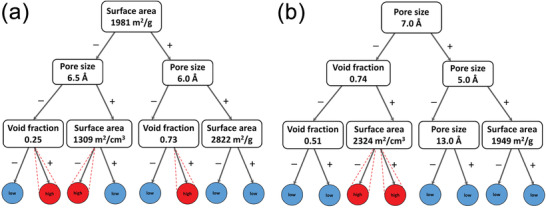
Binary decision tree models of MOFs with a) high CO_2_ capacity (higher than 1 mmol g^−1^) and b) high N_2_ capacity (higher than 0.5 mmol g^−1^). The output nodes referring to low and high gas uptake capacity are highlighted in blue and red color, respectively. Reproduced with permission.^[^ ^78^ ^]^ Copyright 2016, American Chemical Society.

ML has been used to elucidate feature–property relations. Fernandez et al. investigated the relationships between the geometric features of MOFs and their CO_2_ and N_2_ adsorption using an RF model.^[^ ^78^ ^]^ 81 679 MOFs with unique frameworks were collected from the Northwestern University database, from which 16 000 data points were selected as a training set. Five geometric descriptors (dominant pore size, maximum pore size, void fraction, volumetric surface area, and gravimetric surface area) used to train the RF classifier yielded an accuracy >94% for both gases. It identified over 70% and 60% of MOFs known to have high performance for CO_2_ and N_2_ capture, respectively, in a vast search space of ≈65 000 MOFs. They also developed a binary decision tree model to suggest the optimal combination of the five descriptors that enhance the CO_2_ uptake under low pressure, previously only achieved by a sophisticated radial distribution function model (**Figure** **3**). Anderson et al. studied the effects of geometric and chemical features on the prediction accuracy of CO_2_ capture using several ML models.^[^ ^149^ ^]^ The ML methods provided an unbiased approach to evaluating the importance of the descriptors on materials performance, providing useful insight into structure–property relationships. As is often the case, improvement in CO_2_ capture prediction depended strongly on the chemical descriptors, while the absolute values of CO_2_ capture prediction were mostly related to the geometric descriptors. In addition, ML techniques could be used to bypass the complicated first‐principle calculations and predict the partial charges of MOFs, with which the CO_2_ adsorption properties could be accurately calculated.^[^ ^116^ ^]^

Another important application for MOFs is gaseous fuel storage. The effects of different ML algorithms and descriptors on fuel gas adsorption prediction accuracies have been investigated. Pardakhti et al. constructed four ML models of CH_4_ uptake using chemical and crystal structure descriptors.^[^ ^150^ ^]^ The RF model had the best performance, and incorporation of chemical descriptors greatly enhanced prediction accuracy while maintaining computational efficiency. Kim et al. studied the CH_4_ uptake isotherm at a range of temperatures from the isotherm at 298 K using three ML models. Texture features such as surface area and total pore volume, obtained from gas adsorption–desorption experiments, were the major determinants of CH_4_ uptake capacity of MOFs at different temperatures.^[^ ^151^ ^]^ Instead of using experimentally derived features, Gurnani et al. created fingerprints to represent 137 953 hypothetical MOFs.^[^ ^73^ ^]^ They used a series of atomic features describing the coordinating metals (e.g., electronegativity, ionization energy, atomic radii, etc.), and exploited the SMILES strings for the linkers. The speed and generalization ability of the model for CH_4_ uptake capacity of MOFs were analyzed in this report. Wang et al. used the molecular graph (the way the atoms are connected) of MOFs as descriptors and developed a CNN model to predict their CH_4_ adsorption properties.^[^ ^152^ ^]^ This model could reliably recapitulate the properties of the test set so was used to screen a database of 330 000 hypothetical MOFs to discover four MOFs with excellent predicted CH_4_ adsorption ability. Moreover, this model showed good transferability and could be used to predict the CH_4_ adsorption for COF and zeolitic imidazolate framework (ZIF) materials.

**Figure 4:**
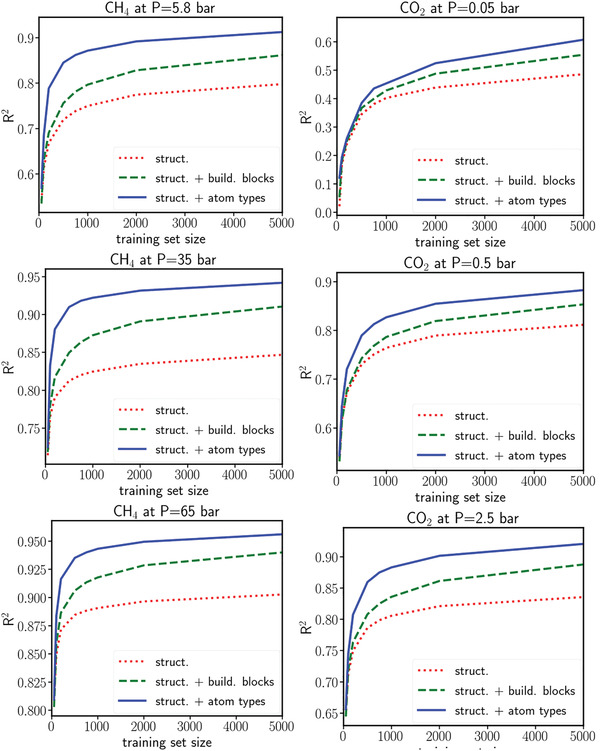
Variation of the *R* ^2^ versus the training set size for (left column) CH_4_ and (right column) CO_2_ under different pressures. Reproduced with permission.^[^ ^155^ ^]^ Copyright 2020, American Chemical Society.

Clearly, feature choice significantly affects the model accuracy. Anderson et al. built a NN model to predict the amount of H_2_ adsorbed at different temperatures and pressures.^[^ ^153^ ^]^ To simplify the model, they used seven features that could be readily obtained by calculations: void fraction, framework density, largest cavity diameter, pore limiting diameter, volumetric surface area, alchemical catecholate site number density, and the epsilon for the interaction of hydrogen with the alchemical sites. This model suggested that a large reduction of pressure (from 100 to 35 bar) only slightly influenced the H_2_ adsorption capacity. Such a reduction of H_2_ pressure could improve safety and compression costs in commercial use. Borboudakis et al. studied CO_2_ and H_2_ adsorption with ensemble learning from three ML models. They represented the structures of the MOFs by encoding the presence or absence of the building blocks (such as organic linker, metal cluster, and functional groups) as a binary parameter.^[^ ^154^ ^]^ Although the accuracy of this method was acceptable, it was not able to predict gas adsorption of MOFs whose linkers or metals were outside of the domain of the training set. To address this, the building block features were substituted by the atom type number density (calculated by the numbers of a particular atom in the MOF unit cell over the unit cell volume) in each structure, and bonds, angles, torsions, and pair interactions were used to represent the elements and connectivity types.^[^ ^155^ ^]^ An RF model was established after training 100 times with randomly selected training sets ranging in size from 50 to 10 000. The effects of three feature families (structural features alone, structural features with MOF building blocks, and structural features with atom type number density) were studied. For CO_2_ and CH_4_ adsorption under the pressures examined, significant improvements were obtained when atom type number density was used (**Figure** **4**). These features allowed the model to be extended to different porous materials such as COFs. Ma et al. trained a DNN model on the H_2_ adsorption data with 13 506 MOFs at 100 bar and 243 K.^[^ ^92^ ^]^ The MOFs were represented by five physical features (void fraction, volumetric surface area, gravimetric surface area, pore limiting diameter, and largest cavity diameter). The good generalizability of this model made it useful for predicting H_2_ adsorption at 130 K as well as being applicable to predicting CH_4_ adsorption under similar conditions. Unsurprisingly, the model showed poor performance when applied to Xe adsorption, indicating the large differences in feature–adsorption relations between fuel gases and inert gases.

The use of solid adsorbents for noble gas adsorption and separation has progressed with the help of ML techniques. Liang et al. built a XGBoost model with seven physical features to predict the Xe/Kr adsorption and selectivity of MOFs.^[^ ^156^ ^]^ Xe is an important propellant used for ion thrusters in spacecraft, therefore, the separation of Xe from Kr is critical for aerospace energy applications. They found that the density, porosity, pore volume, and pore limiting diameter of MOFs are crucial features affecting the Xe/Kr adsorption. Surprisingly, this model could be extended to screen the MOFs for the separation of a CH_4_/CO_2_ mixture.

**Figure 5:**
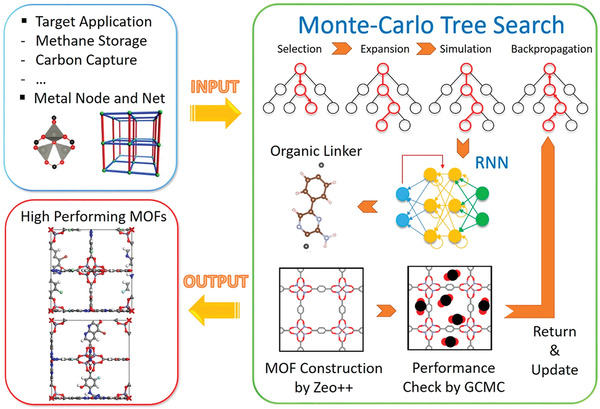
Schematic of an algorithm to design an application‐specific MOF. With given inputs about target application and type of metal node and net, organic linkers were generated by combining the MCTS and RNN. A new MOF constructed by Zeo++ underwent a performance check for the target application and then internal parameters in MCTS were updated. Reproduced with permission.^[^ ^159^ ^]^ Copyright 2020, American Chemical Society.

**Figure 6:**
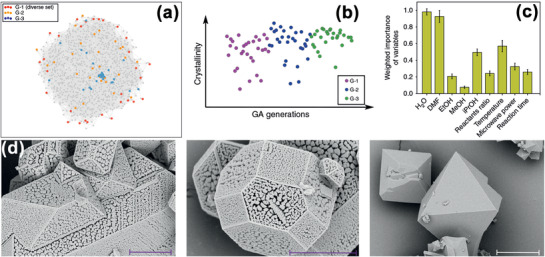
a) Projection from a 9D parameter space to a 2D plane. Grey dots denote the known synthesis conditions. b) Progress in crystallinity in different generations. c) Relative importance of the nine parameters on Cu‐BTC synthesis. d) Scanning electron microscopy images of several Cu‐BTC samples. Scale bars were 5, 4, and 10 µm for the samples shown from left to right, respectively. Reproduced with permission.^[^ ^161^ ^]^ Copyright 2019, Springer Nature.

ML methods can also be used to design MOFs, providing guidance for synthesizing MOFs and other porous materials with bespoke properties. Zhang et al. reported a combined computational approach using a Monte Carlo tree search (MCTS) (an algorithm analogous to reinforcement learning)^[^ ^157^ ^]^ and recurrent neural networks (RNN, a type of NN developed to tackle sequential data)^[^ ^158^ ^]^ to design MOFs for CO_2_ adsorption (**Figure** **5**).^[^ ^159^ ^]^ This approach begins with a given metal vertex, a MOF topology, and the target property (CO_2_ adsorption). An RNN model was trained on 168 130 SMILES strings representing linkers (edges) collected from the ZINC database. The MCTS built a tree in which each node denoted one symbol from the SMILES string by repeating four steps: selection, expansion, simulation, and backpropagation. In the first step, a path from the root (metal node) to a node at *i* level of the current tree was built by choosing the child nodes with maximum upper confidence bound that considered the sum of the target property after *i* simulations. After reaching the leaf of the current tree, child nodes (any valid symbols in SMILES string) were expanded under the nodes at level *i*. Then, the RNN model created the remainder of the strings to simulate a complete linker based on the partial string already built. Using this simulated linker, metal node, and the topology, a MOF was constructed whose CO_2_ adsorption capability was simulated using GCMC. This predicted CO_2_ adsorption value was then backpropagated to the tree and used to update the upper confidence bound on each node. These four steps were repeated iteratively until the string hit the terminal symbol or the maximum length. Several MOFs with high CO_2_ adsorption were thus designed using 10 combinations of metal nodes and topologies extracted from experimental MOFs reported in the literature. Moreover, by applying the topological data analysis, new MOFs with diverse topologies could also be designed. Despite the success of this approach, there can still be difficulties in synthesis or self‐assembly of the MOFs, hence their ability to form stable materials with the expected structures. To address this issue, Collins et al. used a genetic algorithm to optimize and discover MOFs.^[^ ^160^ ^]^ To optimize ZBPs [Zn_2_(1,4‐benzenedicarboxylate)_2_(pyrazine)], they used 28 common functional groups to generate 96 156 hypothetical, stable structures. The materials genome used by the genetic algorithm was the sequence of equivalent sites and their associated functional groups, while the CO_2_ uptake was the fitness function. After genetic algorithm optimization, a 4.8‐fold increase in the CO_2_ uptake was achieved by a new structure. The method was extended to optimize 141 experimentally characterized MOFs, giving rise to 1035 functionalized structures that were predicted to have exceptional CO_2_ uptake (>3 mmol g^−1^ at 0.15 atm and 298 K). Using a different approach, Moosavi et al. attempted to optimize the synthesis conditions and build knowledge on accessible chemistries based on successful and failed synthesis experiments.^[^ ^161^ ^]^ A robot was used to synthesize Cu‐BTC^[^ ^162^ ^]^ (BTC is benzene‐1,3,5‐tricarboxylic acid) by manipulating nine synthesis parameters (**Figure** **6**). Since experimental exploration of these parameters is infeasible, a genetic algorithm was used to accelerate the optimization, with crystallinity, phase purity, and surface area as fitness functions. An RF model was trained to rank parameter importance. It was found that the amount of water and DMF played the largest roles in the synthesis, while the temperature had three times more impact than changing the reactant ratios. Accordingly, a new synthesis could be designed for optimal targets.

There is also increasing interest in the electronic properties and stability of MOFs.^[^ ^130^, ^131^, ^132^ ^]^ Although most MOFs are insulators with bandgaps over 2 eV, conductive MOFs have been discovered by experiments and theoretical simulations.^[^ ^163^, ^164^ ^]^ Because of their high porosity and large surface area, conductive MOFs are largely used for electrochemical energy conversion and energy storage.^[^ ^165^, ^166^, ^167^ ^]^ ML methods have been used to accelerate the screening of large search spaces to discover new conductive MOFs. Despite the large amount of information on MOFs in databases, bandgap information is seldom provided. This is an important characteristic when searching for conductive MOFs. Transfer learning, where the model stores knowledge gained from one property then applies it to solve problems in other properties, was employed to tackle this issue.^[^ ^83^ ^]^ Four ML models (logistic regression, SVM, NN, and RF) were trained using 52 300 inorganic compounds from the Open Quantum Material Database and 45 optimized descriptors. Subsequently, t‐SNE was used to reduce the 45D space to a 2D space, with data points exhibiting some overlap. The authors proposed that this bandgap model could be used to predict the bandgaps of MOFs. To increase the accuracy of prediction, a consensus of predicted bandgaps from four models was used to find those likely to be conductors. From a pool of 2932 MOFs, nine were predicted to be conductive, with six subsequently confirmed as conductive by ab initio calculations. In addition to transfer learning, CNN models were found to accurately predict the bandgap of MOFs. Kernel ridge regression (KRR) models with SOAP fingerprints or composition‐based features predicted bandgaps less accurately than the CNN model.^[^ ^168^ ^]^ The water stability of MOFs is an important property for commercial applications in gas storage, which has also been investigated by ML techniques. ML classifiers have been constructed using features encoding metal electronic properties, linker SMILES strings, molar ratios of the linkers, numbers of O, OH, H_2_O species with respect to metals, culminating in discovery of several MOFs with aqueous stability.^[^ ^169^ ^]^ ML methods were used to avoid the detrimental effects of water on MOF gas adsorption, identifying two water‐stable MOFs by a computational screen of 300 000 MOFs.^[^ ^170^ ^]^

Artifical intelligence approaches have also been applied to design MOF‐based devices. For example, the fabrication of gas (CH_4_) sensors composed of MOF arrays was optimized by a genetic algorithm.^[^ ^171^ ^]^ By optimizing the parameters of the arrays, both the selectivity and the sensitivity of the sensor were significantly enhanced. This study had practical applications in detecting and preventing natural gas leaks in the methane fuel industry.

These examples have exemplified the fact that ML methods can reduce the computational cost and accelerate screening of extremely large MOF spaces. New MOFs with diverse topologies, coordinating metals, and molecular structures for a range of applications have been designed using ML methods.^[^ ^84^ ^]^ These methods can be extended to different frameworks and will widen the applications of MOFs.

### 3.2 Porous Carbon

**Figure 7:**
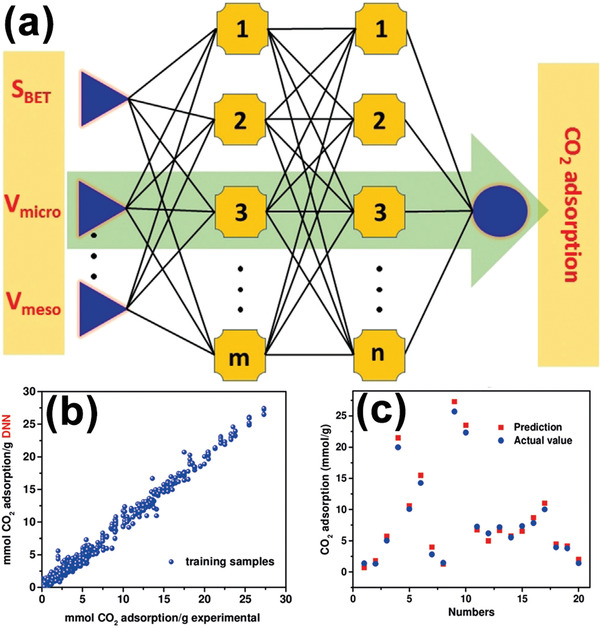
a) Schematic architecture of a DNN model. Inputs were surface area (*S*_BET_), mesopore volume (*V*_meso_), and micropore volume (*V*_micro_), and output was CO_2_ capture capacity. Each line between two nodes represented a weight. By tuning these weights, the input–output relation can be simulated. b) The experimental predicted CO_2_ adsorption versus model predicted CO_2_ adsorption. c) The correlation between 20 experimental CO_2_ adsorption data points and the corresponding model predicted values. Reproduced with permission.^[^ ^174^ ^]^ Copyright 2019, Wiley‐VCH.

Porous carbon is a promising material for gas capture due to its low cost, fast adsorption–desorption kinetics, large surface area and pore volume, and tunable pore structure.^[^ ^172^, ^173^ ^]^ ML approaches can elucidate the relationship between physical properties and gas adsorption ability of carbon.^[^ ^32^ ^]^ Zhang et al. trained a NN model on a set of ≈1000 CO_2_ adsorption data points from literature and experiments (**Figure** **7**).^[^ ^174^ ^]^ The three descriptors that had the most significant effect on the adsorption model were surface area, mesopore volume, and micropore volume. This model accurately predicted CO_2_ adsorption properties of porous carbon materials. Zhu et al. illustrated in detail the effects of different types of pores on CO_2_ adsorption.^[^ ^74^ ^]^ They trained a RF model on 6244 CO_2_ adsorption data points generated for 155 porous carbons and found that increasing the volume of micropores and mesopores had a negative effect on CO_2_ adsorption under low pressure. However, the model indicated that increasing the volume of ultra‐micropores improved CO_2_ adsorption when the pressure increased. To study the selectivity of CO_2_ adsorption, Wang et al. trained a DNN model on experimental data for CO_2_ and N_2_ uptake on porous carbons and concluded that high CO_2_ selectivity could be achieved when the porous carbons possessed moderate micropore (0.4–0.6 cm^3^ g^−1^) and mesopore volumes (0.4–1 cm^3^ g^−1^).^[^ ^175^ ^]^ To further elucidate the effects of porosity on CO_2_ selectivity, a CNN model was built to predict the separation performance of porous carbon, using a CO_2_/N_2_ mixture as a test case.^[^ ^176^ ^]^ The model suggested that the best porous carbon with high CO_2_ adsorption selectivity should have pores with a bimodal size distribution, in which the pore size was in the range of 3–7 nm or less than 2 nm.

The effects of the features of porous carbon on fuel gas adsorption has been studied by ML methods by Zhang et al. who trained a feedforward NN model on the literature data. They used this model to understand the relationships between physical properties of the adsorbent and CH_4_ adsorption.^[^ ^177^ ^]^ Kusdhany et al. also trained an RF model on a dataset of 1745 data points from 68 porous carbons.^[^ ^118^ ^]^ The model showed that pressure and surface area played critical roles in H_2_ uptake capacity prediction, Unlike previous studies, they found that oxygen content was also an important factor in predicting H_2_ uptake, while pore volume had little effect.

These examples indicate how ML can guide the design of highly efficient gas adsorbents and provide a better understanding of the gas adsorption kinetics by highlighting the importance of physical parameters that may have been previously unrecognized. It is expected that ML models will become even more useful tools for designing and optimizing porous materials and uncovering new physical insights about gas adsorption mechanisms when larger and more reliable datasets become available.

### 3.3 Zeolites

Zeolites are microporous crystalline aluminosilicate materials^[^ ^178^ ^]^ with well‐defined cavities and pores that make them very useful for catalysis, adsorption, ion exchange, renewable energy conversion, and water purification.^[^ ^21^, ^178^, ^179^ ^]^ Much effort has been devoted to designing and tailoring zeolites for specific applications.^[^ ^21^, ^180^, ^181^ ^]^ Gas adsorption in zeolites has been more extensively studied by ML techniques than most other porous materials. Pai et al. used ML to optimize the operating conditions for CO_2_ adsorption and selectivity for zeolites, suggesting that this technique could be used to develop zeolites for post‐combustion CO_2_ capture.^[^ ^182^ ^]^ Göltl et al. investigated the CO and NO adsorption of zeolites SSZ‐13 and pentasil zeolite mordenite by a linear regression model.^[^ ^183^ ^]^ The variables in this model were optimized by a genetic algorithm. By analyzing the correlations between the descriptors, they found that the position of the s orbital, the number of valence electrons at the active site, and the HOMO−LUMO gap of the adsorbent had the largest impact on gas adsorption. The reconstruction of the active sites also had a noticeable effect on adsorption. As CO and NO are useful molecular probes in studies of adsorption and conversion of industrial and car exhaust gases (CO, CO_2_, NO_x_) by zeolites, this investigation provides a rational basis for the design of next generation zeolites with improved capacity and activity for the adsorption and conversion of greenhouse gases and toxic exhaust gases.

**Figure 8:**
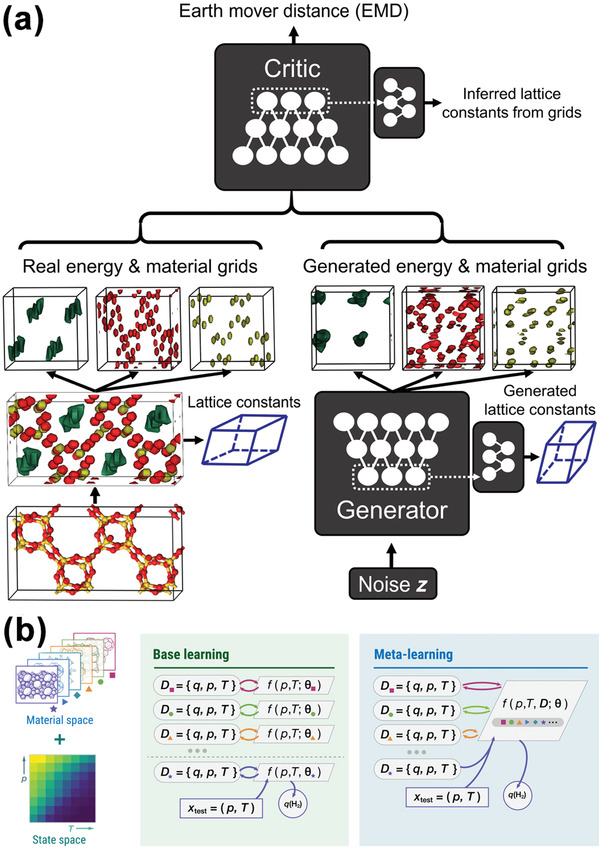
a) Schematic of the generative adversarial NN for zeolite design. Green dots referred to methane potential energy, and material grids indicated silicon (red) and oxygen (yellow) atoms. The energy and material grids of generated zeolites were evolved from Gaussian noise distribution, and the earth mover distance (EMD) between the real and generated energy & material grids was the metric to determine the convergence of training. Periodic padding, feature matching, and lattice constant generating network were added into the “Critic network” to infer rational lattice constants from grids. Reproduced with permission.^[^ ^184^ ^]^ Copyright 2020, American Association for the Advancement of Science. b) Schematic of the meta‐learning technique. Instead of building individual models on different subsets (base learning), meta learning consolidated the prediction of all materials into a single model. Reproduced with permission.^[^ ^186^ ^]^ Copyright 2021, American Association for the Advancement of Science.

State‐of‐the‐art computational techniques have also been used in the zeolite design. Kim et al. implemented a generative adversarial NN to produce novel zeolites for CH_4_ capture (**Figure** **8**a).^[^ ^184^ ^]^ The model was trained on 31 713 zeolites, pairing the positions of oxygen and silicon atoms and the CH_4_ potential energy grids. A total of 121 new porous structures were identified to have the desired heat of adsorption for CH_4_, and this model could be extended to predict the heat of adsorption of other gases on other porous materials including MOFs and COFs. Cho et al. constructed a 3D CNN model on 6500 hypothetical zeolites that exhibited high prediction accuracy for CH_4_ adsorption.^[^ ^185^ ^]^ To enhance the model generalization ability, Sun et al. developed a meta‐learning model to predict H_2_ adsorption for a series of adsorbent materials under a wide range of pressure and temperature.^[^ ^186^ ^]^ Meta learning is a technique that uses ML algorithms to determine the best combination of individual models for a new target (but related to the targets on which the individual models have been trained) with a small amount of training data.^[^ ^187^ ^]^ In the study of gas sorption isotherms, a model showing good performance on one subset (e.g., gas adsorption of a series of materials, or gas adsorption under a range of pressure and temperature) might predict the adsorption poorly on another subset. Therefore, it is necessary to employ meta learning to find correlations between the model performances and the subsets, with which the meta learning model is built to adapt to the gas adsorption dataset generated from multiple materials or under a large range of pressures and temperatures (Figure 8b). Using the meta learning technique, the meta learning model was available for predicting the optimal H_2_ storage temperature under a given pressure for a large variety of adsorbents including all‐silica zeolites, hyper‐crosslinked polymers, and MOFs.^[^ ^186^ ^]^

As gas adsorption and separation often require high pressure, the mechanical properties of zeolites are an important consideration for their commercial use. Evans et al. studied the elastic properties of zeolites using a GBR model and identified important features of SiO_2_ polymorphs that modulate their elastic response.^[^ ^188^ ^]^ Kim et al. used an active learning technique to find the zeolite structures with the highest shear moduli.^[^ ^66^ ^]^ Starting from the International Zeolite Association (IZA) database where only a few zeolites were labeled by their shear moduli, they trained a ML regression model to predict the shear moduli of the rest of the zeolites in the IZA database. Then, they chose the zeolites that were most likely to have good mechanical properties from the test materials via the Bayesian optimization method, labeled their shear moduli by DFT calculation, and returned them to the training set to re‐train the regression model. This process was repeated until the model predictions and the DFT calculations were concordant. 23 novel zeolite structures having excellent shear moduli were discovered using this active learning technique. As with the other porous materials classes, the ZIF examples also illustrate the great potential of ML approaches for design and optimization of zeolite adsorbents.

Apart from property improvement, design of synthesis routes and optimization of synthesis conditions are another important application of ML.^[^ ^189^ ^]^ Most zeolites are generated by hydrothermal synthesis that is controlled by multiple correlated parameters and complex crystallization kinetics.^[^ ^179^ ^]^ This makes it difficult to rationally optimize the synthesis conditions, hitherto relying on trial‐and‐error or theoretical simulation methods to uncover feature‐property relationships. Consequently, ML approaches have been developed to guide the synthesis of zeolites with bespoke properties.^[^ ^190^ ^]^ Daeyaert et al. trained a NN model on a set of 4781 organic structure directing agents, using molecular features as input and the stabilization energy for polymorph A zeolite beta, an important zeolite for enantiospecific catalysis and gas separation, as the predicted property.^[^ ^191^ ^]^ The accuracy of the ML model predictions of stabilization energy was comparable to that from more computationally demanding molecular dynamics simulations. This model was used to search a much larger materials space, and several molecules were identified as structure directing agents in terms of their stabilization energy. These new molecules were potentially useful for the synthesis of polymorph A zeolite beta. Ma et al. reported a ML‐based atomic simulation method to guide design of new Si*_x_* Al*_y_* P*_z_* O_2_H*_y_*_−_ *_z_* zeolites^[^ ^192^ ^]^ that are useful for gaseous fuel adsorption and separation.^[^ ^193^, ^194^ ^]^ They discovered that structure directing agents were important for the formation of micropores for aluminophosphates, silicoaluminophosphates, and pure silica zeolites, while strong alkali was much more important than structure directing agents for the formation of aluminosilicates. Similar ML modeling techniques are being increasingly used in zeolite design and screening.^[^ ^37^, ^190^, ^195^ ^]^

**Figure 9:**
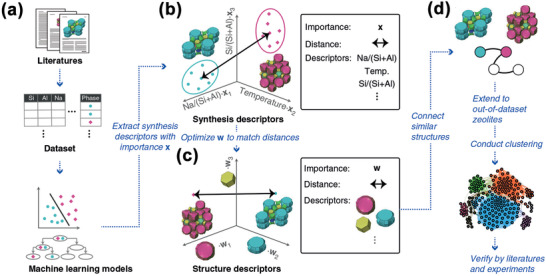
Workflow to link synthesis parameters to structure features in zeolites. a) ML models were constructed from literature data. b) Synthesis parameters mapped the synthesizable domains of zeolites onto a multidimensional phase diagram. x_i_ encoded the importance of each synthesis parameter assessed by the ML models. The synthesis similarity was represented by the distance between the centers of the synthesis conditions for each phase. c) Structure features defined the structural similarity in a multidimensional space representing the presence or absence of building units. d) A network was constructed by connecting structurally similar zeolites based on the structure features. The resulting clustering was verified with data in the literature and experiments. Reproduced with permission.^[^ ^202^ ^]^ Copyright 2019, Springer Nature.

Jensen et al. extracted information on the synthesis of CHA and SFW zeolites from literature using a combination of natural language processing, HTML and XML parsing, and regular expressions.^[^ ^196^ ^]^ A RF model trained on these data indicated the importance of specific synthesis conditions, the Si/Ge molar ratio, the H_2_O/*T* molar ratio (*T* is the TO_4_ tetrahedron in zeolites), and the volume of the organic structure directing agent on zeolite framework design. CHA and SFW zeolites are promising materials for the mitigation of pollutant gases and adsorption of H_2_ and CH_4_. This study provides a pathway to materials with improved clean energy storage and useful environmental remediation properties.^[^ ^197^, ^198^ ^]^ Corma et al. used ML methods to predict synthesis conditions for successful zeolite syntheses,^[^ ^199^ ^]^ a well‐known greenhouse gas adsorbent.^[^ ^200^, ^201^ ^]^ They elucidated the relationships between different synthesis parameters with the performance of Ti‐silicates using a NN model, and used this to optimize the synthesis of the next generation of materials using a genetic algorithm. Specifically, they found that the catalytic performance of the zeolite was enhanced by decreasing the amount of organic modifier while maintaining the OH/Si ratio of ≈0.2. To further illustrate synthesis condition–structure relationships and to provide direction for synthesis of unknown zeolites, Muraoka et al. used an extreme gradient boosting RF model. They extended its prediction space through a similarity network of crystal structures based on structural features and synthesis parameters (**Figure** **9**).^[^ ^202^ ^]^ This model was initially trained on a set of experimental data with synthesis parameters. The good accuracy in predicting the synthesis of zeolites with various structures allowed it to be extended to the prediction of synthesis of zeolites with structures outside the training set. They also generated structure fingerprints for each zeolite and merged them into the feature set. The zeolites were grouped by a k‐means clustering algorithm where the similarity of two zeolites involved both structural and synthesis similarity. With the assistance of this similarity network, the model established optimal conditions for the synthesis of some novel zeolites and thus extended the diversity of the available dataset.

### 3.4 Other Adsorbent Materials

**Figure 10:**
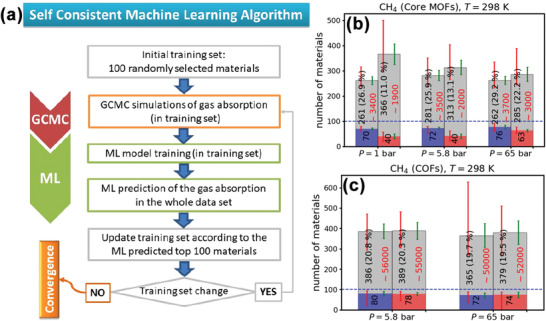
a) Flowchart of the self‐consistent ML approach. From the top of the flowchart, it can be seen that this approach started from randomly picking 100 materials from a database as the initial training set. After labeling by GCMC, a ML model was trained on this training set. The ML model was then used to predict the gas adsorption of all materials in the database, and the top 100 materials would be selected as the new training set until the top 100 materials were the same as the those in the training set. Average results for the b) MOFs and c) COFs obtained from 100 individual runs. Each pair of bars corresponded to calculations with the “high” (blue bar) and the “low” (red bar) accuracy features, respectively. The average number of MOFs and COFs with top performance found during the 100 individual runs was denoted inside these bars. The gray bars show the average number of the total structures included in the final training set. Inside these bars, the number of structures is denoted in black, together with the percentage of the top performing structures found in the final training set. The approximate number of structures required to be randomly selected from the original training set was denoted in red. The red error bar showed the minimum and the maximum value found during the 100 individual runs. The corresponding standard deviation was shown with a green error bar. Reproduced with permission.^[^ ^207^ ^]^ Copyright 2020, American Chemical Society.

COFs (a class of porous polymer framework) have been widely used for H_2_, CH_4_, and CO_2_ storage in clean energy applications.^[^ ^18^ ^]^ Unlike MOFs and ZIFs, COFs are composed entirely of light elements (e.g., H, C, B, O).^[^ ^203^ ^]^ These elements are linked by covalent bonds to form porous structures. High‐throughput COF construction has been facilitated by an evolutionary algorithm, and large COF databases have been constructed.^[^ ^204^ ^]^ However, it takes significant time and resources to explore the large chemical space of COFs with desired properties by traditional calculations (e.g., GCMC). ML approaches have been adopted to accelerate these property predictions. Desgranges et al. created ensemble models by averaging the outputs of the NN models with diverse architectures,^[^ ^205^ ^]^ which were applicable to a broad range of applications such as prediction of CO_2_ adsorption in IRMOF‐1 (Zn_4_O(BDC)_3_, where BDC^2−^ = 1,4‐benzodicarboxylate), H_2_ adsorption by COF‐102, and the separation of methane and ethane by COF‐102 and COF‐108. Optimization of ML model performance was achieved by appropriate choices of algorithms and model descriptors. Yang et al. used a tree‐based pipeline optimization tool (TPOT) in an automated ML platform to analyze the CH_4_ uptake by 403 959 COFs.^[^ ^206^ ^]^ TPOT optimized the model parameters using genetic algorithms, and outperformed other traditional ML models (RF, SVM, etc.). Fanourgakis et al. studied the performance of models of the CH_4_ uptake trained on 69 840 COFs and 4763 MOFs.^[^ ^207^ ^]^ Their results showed that the use of relevant materials features resulted in excellent predictivity for materials properties when models were trained on a small subset of the training data rather than the entire training set (**Figure** **10**). This approach could significantly reduce the computational cost of the construction of the training set using expensive physics‐based methods.

Porous polymer networks (PPNs) are another new type of adsorbent material. They possess a reticular structure with robust covalent bonds of organic linkers. They exhibit superior surface areas and much better stability than MOFs, making them popular choices for gas storage and separation.^[^ ^208^, ^209^, ^210^ ^]^ Pardakhti et al. reported quantitative relationships between the chemical features and CH_4_ uptake of PPNs using RF models trained on 17 846 PPNs.^[^ ^211^ ^]^ Chemical features such as number and type of atoms, electronegativity and degree of unsaturation, played important roles in the CH_4_ uptake under low pressure, while physical features such as surface area and void fraction dominated the adsorption under high pressure. This study highlighted the contributions of surface atoms to gas adsorption that are helpful for adsorbent materials screening and design.

Intermetallics are important materials for gas storage, particularly hydrogen.^[^ ^212^ ^]^ Jäger et al. used a KRR model with local structural descriptors (e.g., SOAP, ACSF) to accurately predict the hydrogen adsorption energy on an Au‐Cu alloy surface. However, construction of local structural descriptors was complex.^[^ ^213^ ^]^ Witman et al. developed a GBT model using the descriptors derived from intermetallic composition only, rather than any structural or hydride information, to predict the log equilibrium pressure of H_2_, lnP_eq_.^[^ ^214^ ^]^ 145 compositional descriptors were used to train the model, and descriptor relevance analysis identified the specific volume per atom for a given composition as most important. Since this has limited physical interpretability, a new descriptor encoding the volume per atom in a crystal was generated and a similar relationship with lnP_eq_ was confirmed. This ML model enabled researchers to predict the hydrogen storage capacity of intermetallic materials from compositional information. ML models have also been employed to predict the CO adsorption energy of a thiolated Au‐Ag nanoalloy. It was found that the CO adsorption largely depended on structural features of the Au‐Ag alloy, and the ML model allowed very fast screening of candidates for further analysis.^[^ ^94^ ^]^

## 4 Summary and Outlook

ML techniques are becoming invaluable for adsorbent materials discovery and design. Coupled with resource‐intensive DFT and GCMC calculations and experiments, ML has robustly and effectively predicted gas uptake, discovered unknown feature–adsorption relations, and optimized synthesis conditions. Many ML models have achieved high accuracy, comparable to first principles calculations, and can elucidate complex feature–property relations efficiently given sufficient reliable data. The use of ML methods allows rapid exploration of large material spaces, provides a rational basis for material design with bespoke properties, and provides new physical insights from large and complex datasets. **Table** **2** summarizes the adsorbents discovered or designed by ML techniques to have substantially improved gas uptake capacity. The application of ML to adsorbent science and engineering is an important step to fast‐track the discovery and optimization of adsorbent materials to address climate change challenges.

**Table 2:** The adsorbent materials with leading gas uptake capacity discovered by ML techniques

Adsorbents	Year of discovery	ML features	ML algorithms	Gas	Results	Note	Ref.
MOF (qtz‐sym‐4‐mc‐Si‐L2)	2019	Topographical features	NN	H_2_	Uptake capacity: 62 g L^−1^ under 100 bar/77 K to 5 bar/160 K	Simulated value. The highest deliverable capacity of H_2_ that can be attained without extreme pressure conditions	[153]
MOF (MFU‐4l (Zn))	2019	Energy histogram, structural features	LASSO	H_2_	Uptake capacity: 47 g L^−1^ under 100 bar/77 K to 5 bar/160 K	Experimental value	[71]
MOF (DUT‐23 (Cu))	2022	Topographical features	GBR	CH_4_	Uptake capacity: 373 cm^3^ (STP) cm^−3^ under 250 bar/120 K to 65 bar/298 K	Experimental value	[215]
MOF (MIL‐47)	2016	Structural features	Genetic algorithm	CO_2_	Uptake capacity: ≈4 mmol g^−1^ at 0.15 atm/298 K 0	Experimental value	[160]
MOF (Al‐PMOF)	2019	Structural features	ML assisted data mining	CO_2_	Uptake capacity: 6 mmol g^−1^ at 2000 mbar/313 K	Experimental value	[170]

The studies summarized in this review show how ML accelerates material development; however, many of the outputs were hypothetical materials. These adsorbent materials may have complicated synthesis procedures or synthesis may not be possible at all. To increase the likelihood of successful synthesis of the adsorbent materials proposed by ML property models, training data and screening scope could also be restricted to materials that have previously been synthesized.^[^ ^89^ ^]^ However, new ML models have now also made significant inroads into predicting the synthesizability of porous materials.^[^ ^202^ ^]^ The results reviewed here also emphasize the importance of close collaboration between computer scientists and experimental experts, both to provide the essential data for training models, but also to allow predictions of ML models to be tested experimentally. Clearly, models are much more useful and generate greater confidence when their predictions are subject to experimental validation. Thus, computational and experimental researchers should work together from the very beginning of projects to establish the ML strategies that achieve materials with optimal properties for a given application.

**Figure 11:**
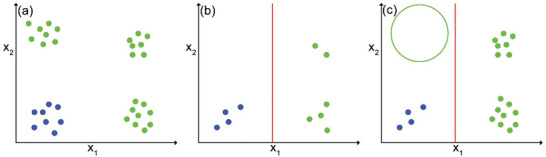
a) The true distribution of the green dots and blue dots in a space, where the green dots are our target. b) On the basis of the initial training set, only a single feature (*x*_1_) may appear important, resulting in a hyperplane perpendicular to the *x*_1_‐axis (red line). c) Once the hyperplane is learnt, the continuous discovery of green dots on the right side of the hyperplane may strengthen the confidence of the model to look for items on the right side of the hyperplane, and thus a correct hyperplane is never determined.

A major challenge limiting the application of ML to the development of adsorbent materials is the size, range, and quality of the dataset. Despite the rise in the number of porous materials databases, collecting calculated and experimental data labeled by target properties (e.g., gas uptake, selectivity, mechanic properties) is expensive and time‐consuming. Therefore, it is important to develop techniques for generating reliable ML models from small samples, especially using high‐throughput and robotic methods. In this review, some cutting‐edge solutions have been described, such as active learning, transfer learning and meta learning, that have been applied to address this issue for adsorbent material studies. However, care must be taken to avoid the attentional learning trap and biases depicted in **Figure** **11**.^[^ ^216^ ^]^ Avoiding this issue requires the involvement of a human operator who, for example, can reduce the rewards in reinforcement learning to force the model to explore new routes, or add different rewards. The limited ability to predict outside the domain of the training data is another limitation, however this will be ameliorated by the increasing availability of data from high‐throughput experiments and computation. Moreover, by identifying an optimally sparse subset of relevant features, overfitting can be avoided when robust models are built using relatively small datasets, and model interpretation simplified. Recently, new techniques based on evolutionary algorithms, such as symbolic learning^[^ ^217^ ^]^ and the sure independence screening and sparsifying operator (SISSO),^[^ ^218^ ^]^ have been developed to generate informative features from large feature pools. The generation of meaningful features is essential to generate robust and predictive models that can usefully guide material development and optimization.

In addition to small sample techniques and feature generation, training data can be expanded by data sources other than databases. Experimental and computational information in the literature can be batch extracted by text mining techniques. Supervised natural language techniques and unsupervised word embedding techniques have been employed to capture the knowledge in the materials science literature.^[^ ^43^, ^89^, ^219^, ^220^ ^]^ However, the sparsity and inhomogeneity of the experimental information from diverse literature sources limits their use for ML model construction. The reproducibility of experimental and computational information is another obstacle to compiling data from heterogeneous sources. Experimental data ideally should be collected by conducting experiments under the same experimental setup and conditions. However, it has been reported replicated syntheses of MOF adsorbent materials is low.^[^ ^221^ ^]^ Some key features of adsorbent materials, such as surface area, are difficult to reproduce because of differences in calculation approaches and ambiguities in molecular structure.^[^ ^222^ ^]^ Eliminating these issues requires authors to provide additional metadata in their publications of synthesis and characterization methods, thereby ensuring the quality and reproducibility of the reported material. Further development and wider use of materials ontologies should also assist in improving reproducibility of syntheses and experimental characterization of adsorbent (and other) materials. Likewise, the reproducibility of the computational information should also be guaranteed by providing open access to the data, input and output files, and the software or codes used for computation.^[^ ^223^ ^]^ In addition, high throughput experiments promise to generate large quantities of data for specific materials systems,^[^ ^32^, ^224^, ^225^ ^]^ but we stress that experimental data on poorly performing materials are also valuable for training the most robust ML models.^[^ ^40^ ^]^ An interesting and very recent development is autonomous laboratories that merge ML techniques with robotics,^[^ ^30^, ^128^, ^226^, ^227^ ^]^ where synthesis and characterization are carried out without human intervention. This is a potentially valuable future solution to collecting high‐quality experimental data on the large scale and autonomously discovering potential adsorbent materials (e.g., porous perovskites,^[^ ^228^ ^]^ porous spinel^[^ ^229^, ^230^ ^]^) with multiple favorable properties (e.g., gases or ions adsorption ability, porosity, selectivity, synthesizability, stability, cost) simultaneously. Further integration of ML, materials science, and engineering will accelerate adsorbent material discovery and find solutions for energy diversification and for combatting climate change.

## Conflict of Interest

The authors declare no conflict of interest.
